# Nécrose gastrique secondaire à un volvulus gastrique compliquant une hernie hiatale

**DOI:** 10.11604/pamj.2020.36.33.23110

**Published:** 2020-05-25

**Authors:** Imen Ben Ismail, Hakim Zenaidi

**Affiliations:** 1 Service de Chirurgie Générale, Centre de Traumatologie et des Grands Brulés Ben Arous, Faculté de Médecine de Tunis, Université Tunis El Manar, El Manar, Tunisie

**Keywords:** Volvulus gastrique aigu, hernies hiatales, nécrose ischémique pariétale de l’estomac, Acute gastric volvulus, hiatus hernias, ischemic gastric wall necrosis

## Abstract

Acute gastric volvulus is a rare condition complicating 4% of hiatus hernias. It is a more or less complete rotation of the stomach around a transverse or longitudinal axis. It can lead to perforations due to ischemic gastric wall necrosis with very poor prognosis (30% of mortality). We here report the case of an 89-year old female patient with chronic bronchopathy, presenting with epigastralgias associated with vomiting which had progressed for 03 days. Physical examination showed febrile patient at 38.5 with dyspnea (SpO2 88% at room air). Her appearence suggested infection, the abdomen was soft, tender at the level of the epigastrium. Laboratory test showed SBI (WBCs= 22000, CRP= 80). Abdominal CT scan showed hiatus hernia associated with significant gastric distension with wall defect in the anterior face of the stomach associated with pneumatosis in the gastric wall. The patient underwent surgery using a median approach, which showed voluminous hiatus hernia containing the greater curvature of the stomach. Distended stomach was restored to its normal intra-abdominal position. The anterior face of the greater tuberosity was the seat of several areas of necrosis. Total gastrectomy with manual oesojejunal anastomosis associated with Roux-en-Y gastric bypass was performed. The postoperative course was complicated by acute respiratory distress syndrome (ARDS). The patient died on day 8 after emergency hospital admission. Gastric volvulus is a rare condition, which can lead to extremely severe consequences. CT scan allows for thoracic lesion assessment and to study stomach vitality. The treatment of choice is based on surgery.

## Image in medicine

Le volvulus gastrique aigu est une situation rare compliquant 4% des hernies hiatales. C'est la rotation plus ou moins complète de l'estomac autour d'un axe transversal ou longitudinal. Il peut conduire à des perforations par nécrose ischémique pariétale de l'estomac, au pronostic très sévère (30% de mortalité). Une patiente âgée de 89 ans, bronchopathe chronique, consultait pour des épigastralgies associées à des vomissements évoluant depuis 03 jours. A l’examen, elle était fébrile à 38.5, dyspnéique (spO2 à 88% à l´air ambiant), ayant un faciès infecté, l’abdomen était souple, sensible au niveau de l’épigastre. A la biologie, SIB (GB= 22000, CRP= 80). Une TDM abdominale a été réalisée montrant une hernie hiatale associée à une importante distension gastrique avec défect pariétal de la face antérieure de l’estomac associé à une pneumatose pariétale gastrique. La patiente a été opérée par voie médiane, l’exploration a trouvé une volumineuse hernie hiatale contenant la grande courbure gastrique. L’estomac distendu a été réintégré en position intra-abdominale. La face antérieure de la grosse tubérosité était siège de plusieurs zones de nécrose Une gastrectomie totale avec anastomose Oeso-jéjunale manuelle sur une anse montée en Y a été réalisée. Les suites opératoires se sont compliquées de SDRA. La malade décédait au 8^e^ jour en réanimation. Le volvulus gastrique est une affection rare, dont l’évolution peut être gravissime. La tomodensitométrie permet de dresser le bilan lésionnel thoracique et d’étudier la vitalité de l’estomac. Le traitement de choix reste chirurgical.

**Figure 1:**
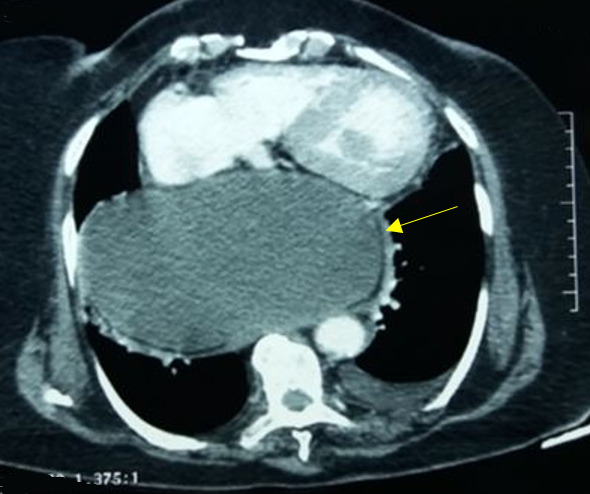
Coupe axiale du scanner thoracique montrant l´estomac hernié en position intrathoracique avec pneumatose pariétale (flèche jaune)

